# Towards a Quantitative Description of Proteolysis: Contribution of Demasking and Hydrolysis Steps to Proteolysis Kinetics of Milk Proteins

**DOI:** 10.3390/foods14010093

**Published:** 2025-01-02

**Authors:** Mikhail M. Vorob’ev

**Affiliations:** A.N. Nesmeyanov Institute of Organoelement Compounds, Russian Academy of Sciences, 28 ul. Vavilova, Moscow 119991, Russia; mmvor@ineos.ac.ru

**Keywords:** proteolysis mechanisms, hydrolysis kinetics, trypsin, β-casein, β-lactoglobulin

## Abstract

The hydrolysis of proteins by proteases (proteolysis) plays a significant role in biology and food science. Despite the importance of proteolysis, a universal quantitative model of this phenomenon has not yet been created. This review considers approaches to modeling proteolysis in a batch reactor that take into account differences in the hydrolysis of the individual peptide bonds, as well as the limited accessibility (masking) for the enzymes of some hydrolysis sites in the protein substrate. Kinetic studies of the proteolysis of β-casein and β-lactoglobulin by various proteolytic enzymes throughout the whole degree of hydrolysis are reviewed. The two-step proteolysis model is regarded, which includes demasking of peptide bonds as a result of opening of the protein structure at the first stage, then hydrolysis of the demasked peptide bonds. To determine the kinetics of demasking, the shift in Trp fluorescence during opening of the protein substrate is analyzed. Two stages of demasking and secondary masking are also considered, explaining the appearance of unhydrolyzed peptide bonds at the end of proteolysis with decreasing enzyme concentrations. Proteolysis of a nanosized substrate is considered for the example of tryptic hydrolysis of β-CN micelles, leading to the formation and degradation of new nanoparticles and non-monotonic changes in the secondary protein structures during proteolysis.

## 1. Introduction

Proteolysis plays a major role in a variety of biological processes and is widely used in biotechnology and food science. Despite the huge number of publications on proteolysis, we do not yet know which stages of this process can be kinetically significant so that they can be used for its quantitative description. A selection of the individual steps in this complex process is typical for biochemistry, and for enzymatic hydrolysis, this is achieved using the Michaelis–Menten equation for non-polymeric substrates with a single specific bond to be cleaved [1,2]. However, it is problematic to use this approach for enzymatic hydrolysis of proteins due to the multiplicity of peptide bonds and the complexity of the spatial structure of the protein substrates [3,4,5]. In this review, we have attempted to formulate new approaches to study proteolysis, which can lead to a quantitative description of this important phenomenon.

Recently, a large number of studies have been published on biopeptides obtained via proteolysis of food proteins, including milk-derived proteins [6,7,8,9]. The chemical structure and physicochemical and functional properties of milk proteins are sufficiently well studied to use them as model substrates in the quantitative study of proteolysis and its modeling. To optimize the proteolytic reaction, it is necessary to know the ratio of the hydrolysis rates of different peptide bonds in the protein being hydrolyzed. Approaches that take into account differences in the hydrolysis of various peptide bonds in proteins, as well as the limited accessibility of some hydrolysis sites to proteases, were considered herein.

## 2. Enzymatic Hydrolysis of Peptide Bonds

The elementary chemical act of proteolysis is the enzymatic hydrolysis of the peptide bond with the formation of a carboxyl group and an amino group: R_1_-CONH-R_2_ + H_2_O → R_1_COOH + NH_2_R_2_. Proteolysis is a complex reaction that includes a large number of elementary reactions involving various polypeptide chains, designated R_1_ and R_2_. A feature of proteolysis is that both the substrate and catalyst are biopolymers. The enzyme is in the form of a globule (the active form of a protein catalyst), while the hydrolyzed substrate is a mixture of various protein substrates, constantly transforming into one another. Initially, the substrate is a protein in its original conformation; it is then converted into a set of polypeptide chain fragments of decreasing length. During the proteolytic reaction, in addition to the hydrolysis of peptide bonds, a decrease in the protein secondary structure occurs, as well as an increase in hydration.

According to Antonov, the author of the first monograph on the chemical aspects of proteolysis [10], before the formation of an enzyme–substrate complex, the attacked peptide chain must be fixed relative to the active site of the proteolytic enzyme (substrate anchoring). Fixation of the attacked peptide bond involves at least two stages. The first, fast stage can be controlled by diffusion, and the second stage is slow, as evidenced by the values of the rate constants of these stages [10,11].

The chemical steps of the peptide bond hydrolysis in low-molecular-weight substrates are well studied for many proteases, and they are especially well studied for serine proteases [12,13]. For the stages of enzyme–substrate complex formation, acylation to form the basic product, and deacylation to form the acidic product, the substrate specificity and the pH profiles of these stages have been studied. However, in the total hydrolysis of peptide bonds in the protein substrates, the different chemical steps are not distinguished, and the effective first- or second-order rate constants (*k*_cat_ or *k*_cat_/*K*_M_) are considered in terms of the Michaelis–Menten parameters *k*_cat_ and *K*_M_ [14,15,16].

Unlike low-molecular-weight substrates with one hydrolyzed bond, for a protein (polypeptide) substrate, there are difficulties in choosing a method for describing the kinetics of its hydrolysis. Indeed, when carrying out proteolysis, it is impossible to hydrolyze some peptide bonds while keeping other peptide bonds unhydrolyzed; therefore, the acts of bond cleavages are interdependent. To obtain quantitative information on the cleavage of peptide bonds, the kinetics of the hydrolysis of individual peptide bonds in the protein substrate should be analyzed, for example, by determining the selectivity parameters [17,18,19]. Selectivity determines the rate of hydrolysis of each individual cleavage site (individual peptide bond) in the protein substrate relative to all cleavage sites in the protein sequence [17]. With this approach, the concentrations of all peptides obtained as a result of hydrolysis of a given site are first summed. Then, this sum—the concentration of the cleavage site products—is plotted against the hydrolysis time, which allows one to analyze the kinetics of hydrolysis of this site. In this way, the selectivity parameters are determined for all specific peptide bonds for a given enzyme in the protein substrate being studied [17,18,19].

The ability to compare numerous experimental data on the hydrolysis of various peptide bonds in protein substrates is provided by the classification proposed by Niemann [20], according to which the specificity of the enzyme includes primary, secondary, and tertiary specificity. The primary specificity corresponds to the interaction with the enzyme of the side chain of the amino acid residue, the carbonyl group of which forms the cleaved bond. The secondary specificity corresponds to the interaction with the enzyme of other amino acid residues of the peptide chain located close to the bond being cleaved. The location of certain amino acids in certain positions of the enzyme active site provides favorable enzyme–substrate binding and catalysis. This can be expressed qualitatively [19,21,22,23] or quantitatively by calculating a set of increments corresponding to the contributions of various amino acid residues at various positions [10,13,24,25,26]. Taking into account the corresponding increments, it is possible to calculate the probability of hydrolysis for any substrate with an arbitrary amino acid sequence [10].

For pepsin, an algorithm for calculating the kinetic constants of hydrolysis of peptide bonds in any peptide with a known sequence of amino acid residues was demonstrated [10]. The values of the hydrolysis rate constants for the attacked peptide bonds were considered the additive function of the contributions of amino acid residues R_i−4_–R_i+5_ located in positions P_+5_–P_−5_ [10,27]. The calculated kinetic parameters for various peptide bonds changed significantly, usually by several orders of magnitude [10,27]. The experimentally determined selectivity parameters for various peptide bonds in the protein substrates also differed significantly [17,18,19,28]. It is clear that the models of proteolysis must consider the dissimilarity of various peptide bonds in the protein substrates, especially the models describing the formation of biopeptides by proteolysis.

This review considers proteolysis by non-immobilized proteolytic enzymes in batch reactors as the simplest proteolytic system amenable to detailed quantitative study. Proteolysis in open-type reactors with immobilized proteases [29,30] and in vitro digestion systems with several enzymes [31,32] are not considered here because their adequate quantitative analysis is more difficult and the quantitative description of proteolysis in such systems is still lacking.

## 3. Milk Proteins as Substrates for Proteolytic Reactions

Proteins from milk whey are used as food ingredients due to their functional and nutritional properties. The major protein in bovine whey is the small globular protein β-lactoglobulin, β-LG (55–60%) [33,34]. Monomeric β-LG is made up of 162 amino acid residues (∼18.3 kDa) and is stabilized by 2 disulfide bonds [34]. Its secondary structure was predicted mainly as a β-sheet (50%) [35]. β-LG and its enzymatic hydrolysates have high functional and nutritional properties. The hydrolysates of β-LG contain many biopeptides that can be used as biologically active additives in therapeutic nutrition and cosmetics [36].

Casein micelles are colloidal complexes of proteins and salts, and their main biological function is to transport sparingly soluble calcium phosphate in liquid form to infants [37]. They have four major protein species, termed α_s1_-, α_s2_-, β-, and κ-caseins [37]. β-CN is the most hydrophobic one within the group of caseins, which constitutes about 45% of the casein of bovine milk [33]. It is a ∼24 kDa single polypeptide chain which consists of 209 amino acid residues [38]. Since β-CN does not contain disulfide bonds, it has no tertiary structure, but some regions of the polypeptide chain have a secondary structure. β-CN has a hydrophobic C-terminus and a hydrophilic negative N-terminal region [39]. Caseins are known to be easily hydrolyzed by proteases due to their conformational flexibility and the abundance of the enzyme-accessible peptide bonds. β-CN is of interest because of its nutritional importance and utility as a drug delivery vehicle [40,41]. The hydrolysates of β-CN, as well as β-LG, are rich in biologically active peptides [42,43].

Due to their low cost and relatively small size, β-CN and β-LG are widely used as protein substrates for proteolytically induced generation of peptides, the number of which is small (several dozen), and their quantity is quite suitable for correct identification and quantification using HPLC-MS methods [44,45,46,47,48,49,50]. Among these studies, it is necessary to highlight those in which quantitative analysis of peptides was carried out not at one point of time but at several points during proteolysis. Such data on the concentration dependences of proteolysis products on the reaction time are suitable for verifying proteolysis models and determining the numerical values of the kinetic parameters. The data for such determinations were published for the proteolysis of β-CN with trypsin [23], engineered trypsin [3,44], chymotrypsin [46], and proteases from *Lactococcus lactis* [5]. Analogical data were published for the proteolysis of β-LG by trypsin [23,45,49], protease from *Bacillus licheniformis* [17,18,19,50], and chymotrypsin [46].

## 4. Proteolysis Models

An analysis of recent studies of proteolysis [51,52,53,54] shows that among the qualitative models of proteolysis, the Linderstrøm–Lang theory [55] proposed in the 1940–1950s of the last century remains popular. The Linderstrøm–Lang theory provides for two extreme cases of the protein degradation under the action of proteases: one-by-one hydrolysis (sequential decay) and zipper hydrolysis (parallel decay).

In the case of the one-by-one mechanism, the limiting step is the opening of the internal peptide bonds of the protein globule, after which most of the peptide bonds can be hydrolyzed. Thus, protein macromolecules are hydrolyzed one after another. A characteristic feature of the one-by-one type is the absence of noticeable intermediate peptides and the presence mainly of the original protein and final peptides [56].

In proteolysis of the zipper type, the hydrolysis of the internal peptide bonds is not limited, and all protein macromolecules participate in the proteolysis process at the same time. In this case, the hydrolysates contain a wide range of intermediate and final peptide fragments [56].

Among the quantitative models of proteolysis, the most popular models are those in which peptide bonds and the rate constants of their hydrolysis are assumed to be the same, and the main variables of the model are the degree of hydrolysis of peptide bonds and the total rate of hydrolysis [57,58,59,60,61,62,63]. These models can be called models of total proteolysis, since the processes of hydrolysis of different sites of hydrolysis (different peptide bonds) are not considered in them. The simplest model of total proteolysis, the exponential model [64,65,66], requires only two parameters for the kinetic description during the whole duration of the process. This model provides a simple mathematical dependence of the rate of hydrolysis on the degree of hydrolysis of peptide bonds in the form of an exponential function. The definition of the different kinetic schemes and the quantification of reaction rate constants for the individual peptide bonds of protein substrate are not required.

The exponential model, like other models of total proteolysis, allows kinetic curves to be described mathematically accurately and the parameters to be calculated, but these parameters do not have a rigorous biochemical background and are often not useful for interpreting experimental patterns. The models of total proteolysis can be used to optimize the conditions for obtaining protein hydrolysates from various proteins, as shown in recent reviews [57,58]. In the models of total proteolysis, all peptide bonds are considered the same [57,58], which is a disadvantage of such models, since this contradicts the experimental data on proteolysis.

**Figure 1:**
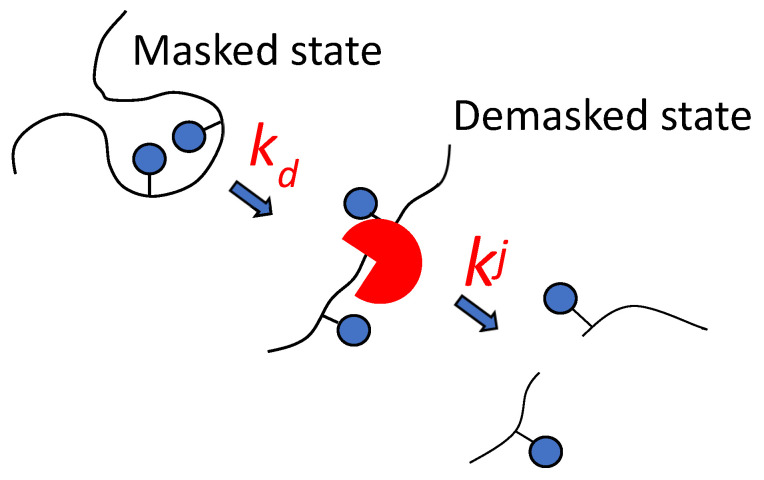
Demasking and hydrolysis steps in the splitting of *j*-th peptide bond are characterized by the rate constants of demasking *k_d_* and hydrolysis *k^j^*.

Considering the complex nature of the proteolysis process, a two-step model of proteolysis was proposed (Figure 1) [4,51]. The new approach proposes that the hydrolyzable site can be hydrolyzed after the demasking step, causing masked peptide bonds to become demasked with the rate constant of demasking (*k_d_*). The hydrolysis of different peptide bonds *j* is realized with different hydrolysis rate constants *k^j^* at the second step—the chemical step of the hydrolysis of demasked peptide bonds. In this step, peptide bonds are hydrolyzed according to their specificity, determined by the amino acid sequence.

The demasking rate constant is determined by the rate of opening of the internal regions of the substrate for subsequent hydrolysis. It was assumed that to describe demasking, it is not the methods of chemical enzymology that are needed, but rather the methods of physical chemistry that characterize changes in the conformation of polypeptide chains over time [4].

This review examines works describing proteolysis according to the scheme shown in Figure 1. In this approach, the rate constants of hydrolysis are different for different peptide bonds, and their hydrolysis may not begin immediately after adding enzyme to the reaction mixture, but after the demasking step.

## 5. Kinetic Evidence of the Existence of Demasking

This section provides experimental kinetic data confirming the presence of a non-hydrolytic stage preceding the hydrolysis of peptide bonds according to Figure 1. The following three processes accompanying proteolysis were studied quantitatively (Table 1):

I. The accumulation of total amino nitrogen during proteolysis, which made it possible to track changes in the rate of hydrolysis during proteolysis;
II. The release of peptide fragments during proteolysis;
III. The cleavage of individual peptide bonds.

**Figure 2:**
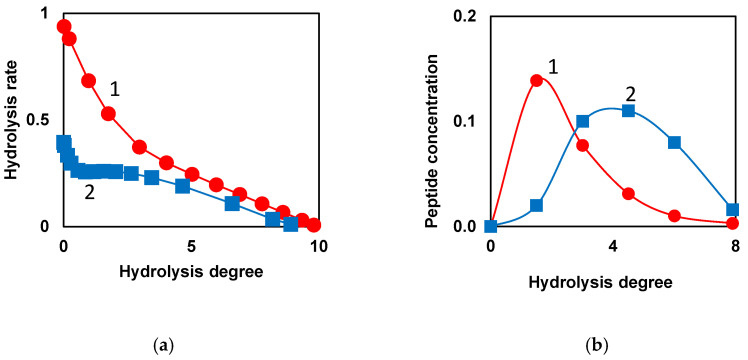
Kinetic patterns indicating the presence of demasking step preceding the hydrolysis of peptide bonds: (**a**) Model dependences of the total hydrolysis rate on hydrolysis degree for low-molecular-weight substrate (•) and protein substrate with initially masked peptide bonds (▪). Demasking and hydrolysis parameters were taken for proteolysis of whole casein by chymotrypsin. Reprinted from [67] with permission from Elsevier © 2013; (**b**) Model release of peptides during proteolysis of β-LG by trypsin [68]. Release of peptide f(9-69) (•) and peptide f(76-91) (▪) according to the model taking into account demasking process [68]. Reprinted from [68]. Copyright © 2023 by the authors. Licensee MDPI, Basel, Switzerland.

When low-molecular-weight substrates with one bond are hydrolyzed, the hydrolysis rate decreases monotonically with increasing hydrolysis time or degree of hydrolysis (curve 1, Figure 2a) [67]. During proteolysis of whole casein by chymotrypsin [67], the overall rate of hydrolysis stops falling in the middle part of the proteolysis process and remains at a relatively high level for some duration of proteolysis (curve 2, Figure 2a). Then, there is a decrease in the rate of hydrolysis, which continues until the end of proteolysis (curve 2, Figure 2a). Similar curves were obtained by extrapolating the kinetics of hydrolysis to zero concentration of the protein substrate when the decrease in the concentration of active enzyme due to inhibition by proteolysis products can be neglected [67]. The explanation for this kinetic feature was demasking [4,67], which was assumed to be generally specific for proteolysis, regardless of the particular protein substrate or proteolytic enzyme. The masking of peptide bonds inside the protein globule or micelle prevents the hydrolysis of a part of the peptide bonds at the beginning of proteolysis. As the steric obstacles are removed as the result of demasking, these peptide bonds begin to hydrolyze, resulting in a shoulder in the curve of the rate of hydrolysis [67].

Interesting proteolysis patterns were noted when modeling the release of individual peptides during proteolysis with the consideration of demasking [68]. The majority of the peptides started to form right after the beginning of proteolysis (curve 1, Figure 2b). However, among the released peptides, one can find a set of peptides (curve 2), the course of formation of which differs significantly from curve 1. During the formation of such peptides, a lag phase can be observed, corresponding to their slow formation at the beginning of proteolysis. The curves of type 2 (Figure 2b) prove the presence of a demasking process preceding the hydrolysis of peptide bonds, due to which the N- and C-terminal groups of such peptides are formed with a lag phase [68].

There are two possibilities for describing proteolysis either in the terms of peptide fragments or individual peptide bonds [68]. Using analytical methods developed in the analysis of the selectivity of peptide bond hydrolysis, it has become possible to analyze and compare the hydrolysis kinetics for the individual peptide bonds of the hydrolyzed protein substrate [68,69]. The concentrations of the individual peptide bonds not hydrolyzed at a certain hydrolysis time were well described by an equation depending on this time, and this equation was derived under the assumption of the existence of a demasking stage preceding the hydrolysis of peptide bonds [68,69]. In turn, the lag phase kinetic patterns were explained when considering the demasking process [52,68,69] (Table 1).

The data collected in Table 1 were obtained by analyzing kinetic curves constructed with discrete points along the course of proteolysis, and this method is very labor-intensive. The importance of demasking processes in the modeling of proteolysis was also noted by using other analytical methods without tedious monitoring of hydrolysis curves [70,71].

## 6. Relationship Between Total Hydrolysis of Peptide Bonds and Conformational Changes in Protein Substrate

When modeling proteolysis, Vorob’ev et al. [72,73] proposed to separate the descriptions of the processes of hydrolysis of peptide bonds and changes in the spatial structure of the protein and its fragments. The peptide bond hydrolysis and changes in protein structure can be viewed simultaneously, allowing one to see how one characteristic relates to another, and vice versa.

Hydrolysis and structure degradation are two sides of the same proteolysis process, but they are different in terms of what is measured and by what methods it is measured. The classical kinetic studies of proteolysis are based on the characterization of the chemical process, the hydrolysis of peptide bonds, which is determined by an increase in the total amino nitrogen *N*(*t*) over time *t* [46,47]. The degree of hydrolysis DH = (*N*(*t*) − *N*(0))/*S*_0_, where *N*(0) and *S*_0_ are the concentrations of amino nitrogen at the beginning of proteolysis and after the complete hydrolysis of all peptide bonds, which is a well-known characteristic of proteolysis [74,75]. To measure changes in the spatial structure of a protein substrate during proteolysis, physicochemical methods, primarily spectroscopic, are required. The possibility of monitoring protein structure changes during course of proteolysis was shown using fluorescence [72,73], infrared (FTIR) [76,77], ultrasonic [71,78,79,80,81], and other physicochemical methods.

The evolution of proteolysis can be depicted graphically, plotting the degree of hydrolysis of peptide bonds on one axis and the degree of degradation of the protein structure on the other. With this representation of the proteolysis process by a curve at these coordinates, inhibition or inactivation of the enzyme does not have a significant effect on it, as shown by the kinetic analysis [67,72]. Significant progress in the development of these ideas and quantification of demasking rate constants was achieved using fluorescence spectroscopy [72,73].

**Figure 3:**
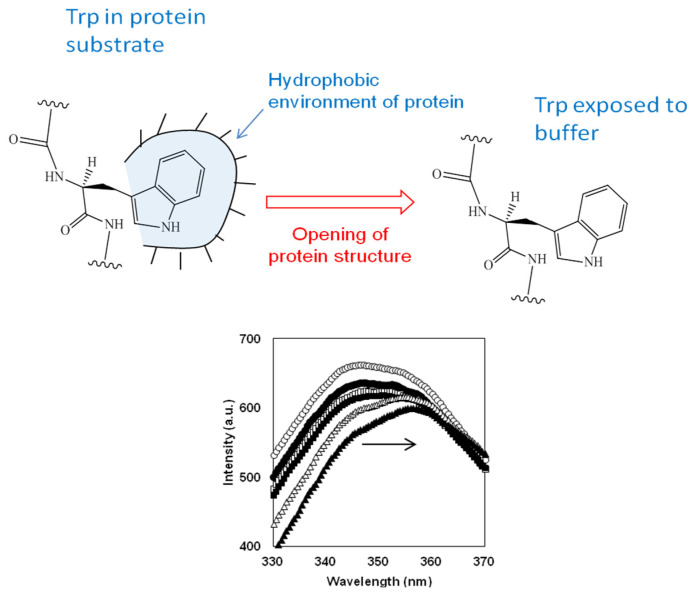
The transfer of tryptophan residues from a hydrophobic environment of surrounding amino acid residues in the protein substrate to a polar media of buffer during proteolysis and corresponding red shift in Trp fluorescence [87]. Reprinted from [87]. Copyright © 2022 by the authors. Licensee MDPI, Basel, Switzerland.

The degree of degradation of the protein structure during proteolysis of β-LG and β-CN with trypsin was determined using fluorescence spectroscopy of tryptophan residues [72]. It was observed that during the course of proteolysis, the positions of emission maxima in the studied spectra were intermediate between those for native protein and free L-Trp amino acid, where the solvent polarity around Trp residues is the lowest and the highest possible values, respectively. It is known that the position of the maximum fluorescence emission of tryptophan residues is sensitive to their closest environment [82,83]. The opening of a protein substrate during proteolysis leads to a corresponding increase in the polarity of amino acid residues that were initially in a relatively hydrophobic environment of protein substrate, as shown in Figure 3. An increase in the polarity of amino acid residues such as tryptophan, which have the maximum fluorescence among aromatic amino acid residues, leads to a red shift of their fluorescence, i.e., the fluorescence emission spectra shift toward higher wavelengths (Figure 3). Thus, the degradation of the structures of protein substrate can be recorded for any proteolysis time *t* by measuring the wavelength *λ*_max_(*t*) at the maximum tryptophan emission [72]. An alternative to monitoring proteolysis through measurement of wavelength shift is to monitor changes in fluorescence intensity during proteolysis [84,85,86].

For the evaluation of the fluorescence shift, a simple parabolic approximation *I*(*λ*) = *aλ*^2^ + *bλ* + *c* of the top part of the fluorescence spectrum *I*(*λ*) was used within the short interval of the wavelength around the peak maximum *λ*_max_ = −*b*/2*a* [72]. The position of the fluorescence maximum (*λ*_max_) as a function of proteolysis time represents the increasing dependence for the proteolysis of β-LG and β-CN [72].

It was proposed that the opening of protein substrate, as measured by its Trp fluorescence, can be used to assess the state of demasking [72]. Thus, the fluorescence shift made it possible to determine the degree of demasking of peptide bonds, i.e., the portion of those not yet hydrolyzed peptide bonds that are accessible for enzymatic attack [72]. The degree of demasking was calculated as (*λ*_max_(*t*) − *λ*^0^)/Δ*λ*, where Δ*λ* represents the maximum increase in *λ*_max_ during proteolysis, and *λ*^0^ is the wavelength of the maximum emission for the native protein at the beginning of proteolysis (*t* = 0) [72].

**Figure 4:**
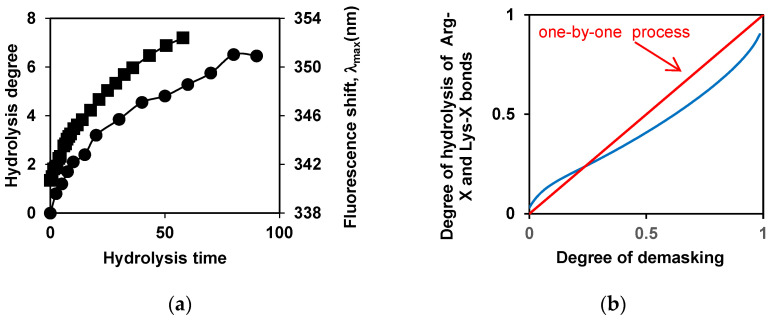
The interdependence of the degree of demasking and the degree of hydrolysis for proteolysis of β-LG by trypsin [88]: (**a**) Dependences of the degrees of hydrolysis (•) and demasking (▪) on the time of hydrolysis; (**b**) Dependences of the degree of demasking and degree of hydrolysis of enzyme-specific peptide bonds (Arg-X + Lys-X bonds) against each other. Experimental dependence (blue) and theoretical dependence (red) for one-by-one mechanism of proteolysis. Reprinted from [88]. Copyright © 2019 by the authors. Licensee MDPI, Basel, Switzerland.

An example of the interdependence of the degree of demasking and the degree of hydrolysis is shown for the tryptic proteolysis of β-LG in Figure 4. Figure 4a shows the time dependences of these proteolysis characteristics, and Figure 4b shows these two characteristics against each other, with time excluded.

Based on the theoretical considerations, it follows that the straight line in the coordinates degree of demasking—the degree of hydrolysis corresponds to one-by-one proteolysis [51,56]. The comparison of the curves shows that β-LG proteolysis by trypsin is close to this type of proteolysis (Figure 4b). It turned out that the course of proteolysis of β-CN by trypsin depends on the state in which it was before hydrolysis—in micellar form or in molecular form. The proteolysis of molecular β-CN with trypsin was carried out at β-CN concentrations below the critical micelle concentration (CMC) [88], although in this case, some proteolysis products are prone to aggregation, as in the case of the proteolysis of micellar β-CN [89].

Comparing the proteolysis of β-CN and β-LG at the beginning of the process, it was shown that for β-CN in the non-micellar form, *λ*_max_ can first decrease, which corresponds to the aggregation of large hydrolysis products, and then increase [88]. This differs from the monotonous increase in *λ*_max_ throughout the entire proteolysis process for β-LG. Thus, the kinetic curves describing demasking of protein substrates are indeed different, and the largest differences can be found at the beginning of proteolysis [88].

## 7. Two-Step Proteolysis Model

For the simulation of connection of the growth in peptide bond hydrolysis with demasking progress, a simple two-step proteolysis model was proposed [4]. This basic model includes only two subsequent steps, corresponding to demasking and hydrolysis processes [4,72,73]. It was applied to the proteolysis by trypsin [4,72,73,88] and by chymotrypsin [67] in the framework of the primary specificity of these enzymes. For trypsin, the basic two-step model includes a set of hydrolysis rate constants for Lys-X and Arg-X peptide bonds and only one demasking rate constant *k_d_*. Among the various hydrolysis rate constants *k^j^* for different peptide bonds *j*, the value *k_h_* is the largest of them. The model also includes restrictions in the hydrolysis of some peptide bonds, describing them using the parameters *m* and *n*. In this model, *m* is the initial degree of masking (the portion of initially masked peptide bonds). The parameter *n* is the portion of peptide bonds that are enzyme-specific but resistant to hydrolysis [73,88]. The two-step model allowed for fitting of experimental curves in the coordinates of the degree of demasking—the degree of hydrolysis (Figure 4b)—which made it possible to determine the parameters *k_d_*/*k_h_*, *m* and *n* [73,88]. In [72], it was assumed that *n*=0, while in [73,88], all three parameters were varied.

The following values of the ratio of demasking and hydrolysis rate constants *k_d_*/*k_h_* were obtained: 0.050 (β-LG) and 0.033 (β-CN), which indicates that the demasking rate constant is at least an order of magnitude lower than the rate constant of hydrolysis of the most specific peptide bond [73,88]. It was shown that demasking can initially limit the hydrolysis of approximately half of the peptide bonds (*m* = 0.56 and 0.61 for β-LG and β-CN, respectively).

The basic two-step model considers proteolysis throughout its entire length from the beginning to the end, and the model has only one parameter characterizing the demasking stage. It was unexpected that during the proteolysis of such different substrates as β-CN and β-LG, not very different values of the ratio of the demasking and hydrolysis rate constants were obtained [73,88]. The introduction of the additional stage of demasking and secondary masking improved the modeling accuracy, as follows from the next section [87,90].

## 8. Two-Stage Demasking and Secondary Masking

In the previous section, the kinetics of proteolysis was considered using the basic two-step model with uniform demasking of all hydrolysis sites of protein substrate, and only one demasking rate constant was used to quantify this process. This section considers a more complex model of proteolysis, under the assumption that some sites, as before, can be hydrolyzed after one demasking stage, while other sites need to undergo two demasking stages to be hydrolyzed [90]. In addition, it was assumed that after the first stage of demasking, some sites can be irreversibly masked again; thus, if they have no time to be hydrolyzed, these sites may remain unhydrolyzed [87]. For such sites (Table 2), the rate constant of secondary masking is not equal to zero (*k_m_* ≠ 0) [87].

Table 2 shows four kinetic schemes corresponding to the models with or without two demasking stages and with or without the stage of secondary masking. The analysis of these schemes gives equations for the concentrations of the products *N^j^*(*t*) formed by the hydrolysis of bond *j* at any hydrolysis time *t* (Table 2). Which of the schemes should be applied to the hydrolysis of certain peptide bonds was demonstrated using the example of the proteolysis of β-LG by trypsin [87,90].

**Figure 5:**
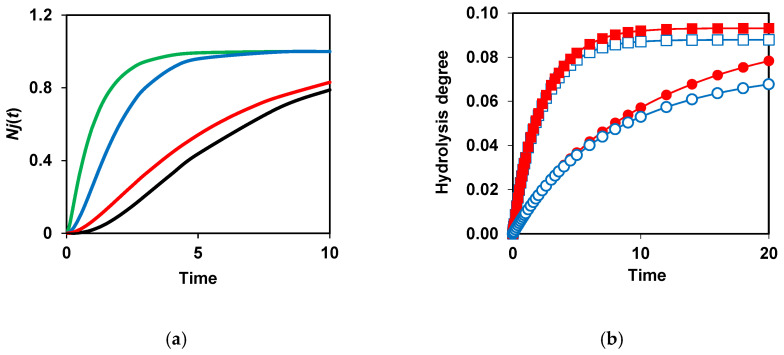
Modeling of proteolysis kinetics taking into account two-stage demasking and secondary masking [87,90]: (**a**) Kinetics of bond hydrolysis depending on the parameters of demasking of these bonds bonds at *k^j^* = 1. One-stage demasking at $k_{d}^{f}$ = 10 (green), $k_{d}^{f}$ = 1 (blue), and $k_{d}^{f}$ = 0.2 (red). Two-stage demasking at $k_{d}^{f}$ = 1 and *k_d_* = 0.2 (black); (**b**) Incomplete hydrolysis of peptide bonds due to their secondary masking. Comparison of proteolysis without secondary masking at *E* = 2 (■), 0.4 (●) and proteolysis with secondary masking (*k_m_* = 0.05) at *E* = 2 (□), 0.4 (○). Reprinted from [87,90]. Copyright © 2020 and 2023 by the authors. Licensee MDPI, Basel, Switzerland.

The need to demask peptide bonds before their hydrolysis causes the appearance of a lag phase in the functions *N^j^*(*t*) [90]. Figure 5a shows how decreasing *k_d_* and adding a second demasking stage results in more pronounced lagged kinetics. Theoretically, this effect should be mostly noticeable with two-stage demasking and low values of the demasking rate constants [90]. It should be noted that in the literature on proteolysis, there was no explanation of the phenomenon of the lag-phase kinetics and the reasons for its occurrence. The consideration of the demasking of peptide bonds during proteolysis allowed us to fill this gap [68,90].

An important innovation was the consideration of the proteolysis schemes that take into account not only the primary masking caused by the initial structure of the protein substrate but also the secondary masking that occurs during the formation of intermediate proteolysis products [87]. Considering secondary masking makes it possible to explain why, at insufficient enzyme concentrations (small *E*/*S* ratios), it is often not possible to achieve complete hydrolysis of the specific peptide bonds even at long hydrolysis times. This phenomenon was observed experimentally for the proteolysis of various enzyme–substrate pairs [91,92,93,94,95,96]. In particular, the peptides with unhydrolyzed specific peptide bonds were found in the peptide aggregates of intermediate proteolysis products of whey proteins [91,92].

Figure 5b shows that there is no difference in the final degrees of hydrolysis at *k_m_* = 0 at different enzyme concentrations. The difference appears if *k_m_* is not zero, i.e., when there is secondary masking. The number of unhydrolyzed bonds at the end of proteolysis increases with decreasing enzyme concentrations at a constant substrate concentration [87] (Figure 5b). In general, to accelerate the hydrolysis of peptide bonds and their more complete cleavage, it is necessary to increase the rate of demasking and reduce secondary masking.

The effects of secondary masking on the kinetics of the hydrolysis of peptide bonds, which are demasked by one-stage demasking, are described by Equation (3) (Table 2). From this equation, it follows that the maximal concentration of the products resulting from hydrolysis of *j*-th bond at the end of proteolysis is *N*_0_*k^j^E*/(*k_m_* + *k^j^E*). It is not equal to *N*_0_, but it depends on the proteolysis conditions, i.e., the concentration of enzyme *E*. At *k_m_* ≠ 0, the decrease in the enzyme concentration leads to a decrease in the term *k^j^E*/(*k_m_* + *k^j^E*). As *k_m_* increases, the apparent hydrolysis rate constant *k_m_* + *k^j^E* should increase, while the maximal concentration of the products resulting from the hydrolysis of the *j*-th bond should decrease. Thus, the use of Equation (3) explains why, under some proteolytic conditions, some peptide bonds are not completely hydrolyzed at the end of proteolysis. These patterns can be observed, for example, for the hydrolysis by acid protease from *Bacillus licheniformis* of peptide bonds 77 and 88 located at the surface of β-LG. The hydrolysis of these peptide bonds was very limited in experiments carried out at different substrate and enzyme concentrations at a constant *E/S* ratio [17,68]. The importance of taking into account secondary masking was also recently demonstrated for the hydrolysis of α-LA by pepsin [52].

A simulation of peptide release during the proteolysis of β-LG by trypsin was demonstrated using the original method with consideration of the demasking process without secondary masking [68]. The simulation of the kinetics of proteolysis was carried out with some simplifications regarding the mechanism of the process and the calculation procedure. It was assumed that there was no secondary masking (*k_m_* = 0), the length of the resulting peptide fragments was limited by two enzyme-specific peptide bonds inside them that could be further hydrolyzed, and the formation of minor peptide fragments was not considered. However, good agreement between the calculated and experimental concentration curves of the peptide release during proteolysis was demonstrated [68]. This agreement was achieved by taking into account the one-stage and two-stage demasking processes.

## 9. Proteolysis of Nanosized Aggregates and Micelles

The consideration of the proteolysis of nanosized protein substrates is much more complex than soluble protein macromolecules and requires the use of additional analytical methods and new methodological approaches. The main unresolved issue is the determination of the mechanism of enzymatic attack of protein particles, which includes coupled penetration of the enzyme into the depths of the protein particle and the degradation of the surrounding protein matrix, freeing up space for the movement of the enzyme. It is also unclear how different the hydrolysis rate constants in the compact protein particles may be from the hydrolysis rate constants traditionally determined in the buffer solutions of well-hydrated protein substrates.

As a simple example of proteolysis with the participation of the nanosized protein structure, tryptic hydrolysis of micellar β-CN at a concentration above the CMC was studied [89]. The native and hydrolyzed soap-like β-CN micelles were found to be good nanoscale objects that could be analyzed quantitatively using atomic force microscopy (AFM) [97]. However, the presence of a large number of nanoparticles of different sizes with different degrees of degradation of the polypeptide chain made it possible to build only a simple quantitative model [89].

According to AFM, FTIR, and static light scattering data, during proteolysis, not only the degradation of the original β-CN micelles occurs, but also the parallel formation of new nanoparticles and their further degradation [89,97,98]. These new nanoparticles are denser than the original micelles, and the content of secondary protein α-, β-structures in them is comparable to those in the original β-CN micelles. Due to this, during proteolysis at low enzyme concentrations, the content of the secondary protein structures first decreases due to the hydrolysis of original micelles, then increases due to the formation of these new nanoparticles, and then slowly decreases as they are hydrolyzed [89].

The degradation kinetics of β-CN micelles by trypsin were described using analytical functions in the case of modeling of the proteolytic system using linear differential equations [89]. This process was also described using numerical simulation methods with nonlinear differential equations [99]. In this case, micelles, nanoparticles of various sizes, and their aggregates, as well as changes in the concentration of the active enzyme during proteolysis, were considered [99].

The degradation of milk casein micelles by proteases is an even more complex process than the degradation of β-CN micelles, which is due to the complexity of the structure of milk casein micelles [100,101]. To our knowledge, the proteolysis of milk casein micelles has not yet been described in the framework of chemical kinetics using the rate constants of the stages of the degradation process.

## 10. Conclusions

The main idea of the approach discussed here is to separate the proteolysis process into two main steps. The first one is demasking, which is associated with the destruction of protein structure and the opening of peptide bonds for enzymatic attack. The second step, the truly chemical step, is the hydrolysis of the peptide bond itself. There is no additional division of the hydrolysis step into several stages, but the demasking step, on the contrary, can be divided into two stages. Among the complications of the model, secondary masking was introduced, which explains why, depending on the different enzyme concentrations, some peptide bonds may remain unhydrolyzed at the end of the proteolysis process. The analysis of demasking requires the use of unconventional enzymological methods such as spectroscopic methods for assessing the opening of the protein substrate. The use of the methods of physical chemistry and computer modeling of kinetics is also necessary for modeling the enzymatic degradation of nanosized protein structures such as protein micelles.

The proposed approach of describing proteolysis introduces new concepts such as the demasking process, the demasking kinetics, the demasking rate constants at various stages of demasking, and secondary masking. To determine the numerical values of the demasking rate constants, fluorescence spectroscopy can be used, which made it possible to monitor the fluorescence shift of Trp residues during proteolysis. It is reasonable to use this methodology in proteolysis studies where it is necessary to establish the exact course of the hydrolysis of individual peptide bonds in the protein substrate. For example, to find the conditions at which bioactive peptides are formed throughout the proteolysis process, one needs to know the demasking and hydrolysis rates for different peptide bonds in the studied protein substrate. The developed approach can also be useful for optimizing the production of protein hydrolysates in terms of the increasing the demasking rate and reducing secondary masking.

## Figures and Tables

**Table 1:** Kinetic patterns indicating the presence of a stage preceding the hydrolysis step.

	Substrate	Enzyme	Process	Observed Feature	Reference
I. Hydrolysis of all peptide bonds	Whole casein	Chymotrypsin	Growth of total amine nitrogen during proteolysis	Non-monotonous decrease in hydrolysis rate, Figure 2a	[67]
II. Release of peptide fragments	β-CN	Trypsin	Release of peptide Gly_203_-Val_209_	Presence of lag phase in the release of this peptide, Figure 2b	[4]
	β-LG	Trypsin	Release of peptides: Ile_84_-Lys_91_, Thr_125_-Lys_135_, Phe_136_-Lys_138_	Presence of lag phase in the release of these peptides, Figure 2b	[68]
III. Hydrolysis of individual peptide bonds	β-LG	Protease from *Bacillus licheniformis*	Cleavage of individual peptide bonds: Glu_51_-Gly_52_, Glu_65_-Cys_66_, Glu_112_-Pro_113_, Glu_114_-Gln_115_, Glu_131_-Ala_132_, Asp_11_-Ile_12_, Asp_28_-Ile_29_, Asp_33_-Ala_34_, Asp_85_-Ala_86_, Asp_96_-Thr_97_, Asp_129_-Asp_130_, Asp_137_-Lys_138_	Hydrolysis of these bonds corresponds to equation for sequential demasking and hydrolysis kinetics	[69]
	α-lactalbumin (α-LA)	Pepsin	Cleavage of individual peptide bonds: Phe_9_-Arg_10_, Leu_23_-Pro_24_, Phe_80_-Leu_81_, Asp_83_-Asp_84_	Presence of lag phase in the hydrolysis of these peptide bonds	[52]

**Table 2:** Modeling of proteolysis with consideration of two-stage demasking and secondary masking.

Type of the Proteolysis Process	Kinetic Scheme and Equation for the Concentration of the Products of the Hydrolysis of *j* Bond ^1^
	$B_{m}^{j}\overset{k_{d}^{f}}{\to }B_{d}^{j}\overset{k^{j}}{\to }N^{j}$
One-stage demasking without secondary masking	$N^{j}(t)=N_{0}\left[1-\frac{k^{j}e^{-k_{d}^{f}t}}{\left(k^{j}-k_{d}^{f}\right)}+\frac{k_{d}^{f}e^{-k^{j}t}}{\left(k^{j}-k_{d}^{f}\right)}\right]$ (1)
	$B_{m}^{j}\overset{k_{d}^{f}}{\to }B_{d}^{j}\overset{k_{d}}{\to }B_{dd}^{j}\overset{k^{j}}{\to }N^{j}$
Two-stage demasking without secondary masking	$N^{j}(t)=N_{0}\left[1-\frac{k_{d}k^{j}e^{-k_{d}^{f}t}}{\left(k_{d}-k_{d}^{f})(k^{j}-k_{d}^{f}\right)}-\frac{k_{d}^{f}k^{j}e^{-k_{d}t}}{\left(k_{d}-k_{d}^{f})(k_{d}-k^{j}\right)}-\frac{k_{d}^{f}k_{d}e^{-k^{j}t}}{\left(k^{j}-k_{d}^{f})(k^{j}-k_{d}\right)}\right]$ (2)
	Bmj→k1E km Bdj↓Bmm→kjENj
One-stage demasking and secondary masking	$N^{j}(t)=N_{0}\frac{k^{j}E}{k_{m}+k^{j}E}\left[1-\frac{(k_{m}+k^{j}E)e^{-k_{1}Et}}{k_{m}+k^{j}E-k_{1}E}+\frac{k_{1}Ee^{-(k_{m}+k^{j}E)t}}{k_{m}+k^{j}E-k_{1}E}\right]$ (3)
	Bmj→k1E km Bdj↓Bmm→k2EBddj→kjEN2j
Two-stage demasking and secondary masking	$N^{j}(t)=N_{0}\frac{k_{2}E}{k_{m}+k_{2}E}\left[1-\frac{(k_{m}+k_{2}E)k^{j}Ee^{-k_{1}Et}}{\left(k_{m}+k_{2}E-k_{1}E)(k^{j}E-k_{1}E\right)}-\frac{k_{1}Ek^{j}Ee^{-(k_{m}+k_{2}E)t}}{\left(k^{j}E-k_{m}-k_{2}E)(k_{1}E-k_{m}-k_{2}E\right)}-\frac{(k_{m}+k_{2}E)k_{1}Ee^{-k^{j}Et}}{\left(k^{j}E-k_{1}E)(k^{j}E-k_{m}-k_{2}E\right)}\right]$ (4)

^1^ $B_{m}^{j}$ is the jth peptide bond in the intact protein substrate, $B_{d}^{j}$ is the jth peptide bond in the partially demasked state, $B_{dd}^{j}$ is the jth peptide bond in the completely demasked state, *B_mm_* is the peptide bond in the secondary masked products, *E* is the free enzyme, *N*^j^ is the products of hydrolysis of jth peptide bond, $k_{d}^{f}$ and *k_d_* are the rate constants for the first and second stages of demasking in Equations (1) and (2), *k^j^* is the hydrolysis rate constant for jth peptide bond in Equations (1) and (2), $k_{1}E$ and $k_{2}E$ are the rate constants for the first and second stages of demasking in Equations (3) and (4), *k^j^E* is the hydrolysis rate constant for jth peptide bond in Equations (3) and (4), *k_m_* is the rate constant of secondary masking. A detailed analysis of the kinetic schemes is provided in [87,90].

## Data Availability

No new data were created or analyzed in this study. Data sharing is not applicable to this article.

## References

[1] Fersht A. (1984). Enzyme Structure and Mechanism.

[2] Cornish-Bowden A. (1995). Fundamentals of Enzyme Kinetics.

[3] Vorob’ev M.M., Dalgalarrondo M., Chobert J.-M., Haertle T. (2000). Kinetics of β-casein hydrolysis by wild-type and engineered trypsin. Biopolymers.

[4] Vorob’ev M.M. (2009). Kinetics of peptide bond demasking in enzymatic hydrolysis of casein substrates. J. Mol. Catal. B.

[5] Muñoz-Tamayo R., De Groot J., Wierenga P.A., Gruppen H., Zwietering M.H., Sijtsma L. (2012). Modeling peptide formation during the hydrolysis of β-casein by *Lactococcus lactis*. Process Biochem..

[6] Du Z., Li Y. (2022). Review and perspective on bioactive peptides: A roadmap for research, development, and future opportunities. J. Agric. Food Res..

[7] Tacias-Pascacio V.G., Morellon-Sterling R., Siar E.-H., Tavano O., Berenguer-Murcia Á., Fernandez-Lafuente R. (2020). Use of Alcalase in the production of bioactive peptides: A review. Int. J. Biol. Macromol..

[8] Bo W., Chen L., Qin D., Geng S., Li J., Mei H., Li B., Liang G. (2021). Application of quantitative structure-activity relationship to food-derived peptides: Methods, situations, challenges and prospects. Trends Food Sci. Technol..

[9] Chalamaiah M., Yu W., Wu J. (2018). Immunomodulatory and anticancer protein hydrolysates (peptides) from food proteins: A review. Food Chem..

[10] Antonov V.K., Tang J. (1977). New data on pepsin mechanism and specificity. Acid Proteases: Structure, Function, and Biology. Advances in Experimental Medicine and Biology.

[11] Vorob’ev M.M., Vitt S.V., Belikov V.M. (1987). Kinetic description of proteolysis. Part 3. Total kinetics of peptide bonds hydrolysis in peptide mixtures. Nahrung-Food.

[12] Hedstrom L. (2002). Serine protease mechanism and specificity. Chem. Rev..

[13] Schellenberger V., Braune K., Hofmann H.J., Jakubke H.D. (1991). The specificity of chymotrypsin. A statistical analysis of hydrolysis data. Eur. J. Biochem..

[14] Salami M., Yousefi R., Ehsani M.R., Dalgalarrondo M., Chobert J.-M., Haertle T., Razavi S.H., Saboury A.A., Niasari-Naslaji A., Moosavi-Movahendi A.A. (2008). Kinetic characterization of hydrolysis of camel and bovine milk proteins by pancreatic enzyme. Int. Dairy J..

[15] Olsen K., Otte J., Skibsted L.H. (2000). Steady-state kinetics and thermodynamics of the hydrolysis of β-lactoglobulin by trypsin. J. Agric. Food Chem..

[16] Shi D., He Z., Qi W. (2005). Lumping kinetic study on the process of tryptic hydrolysis of bovine serum albumin. Process Biochem..

[17] Butre C.I., Sforza S., Gruppen H., Wierenga P.A. (2014). Introducing enzyme selectivity: A quantitative parameter to describe enzymatic protein hydrolysis. Anal. Bioanal. Chem..

[18] Butré C.I. (2014). Introducing Enzyme Selectivity as a Quantitative Parameter to Describe the Effects of Substrate Concentration on Protein Hydrolysis. Ph.D. Thesis.

[19] Deng Y., Gruppen H., Wierenga P.A. (2018). Comparison of protein hydrolysis catalyzed by bovine, porcine, and human trypsins. J. Agric. Food Chem..

[20] Niemann C. (1964). Alpha-chymotrypsin and the nature of enzyme catalysis. Science.

[21] Gershon P.D. (2014). Cleaved and missed sites for trypsin, Lys-C, and Lys-N can be predicted with high confidence on the basis of sequence context. J. Proteome Res..

[22] Suwareh O., Causeur D., Jardin J., Briard-Bion V., Le Feunteun S., Pezennec S., Nau F. (2021). Statistical modeling of in vitro pepsin specificity. Food Chem..

[23] Deng Y., van der Veer F., Sforza S., Gruppen H., Wierenga P.A. (2018). Towards predicting protein hydrolysis by bovine trypsin. Process Biochem..

[24] Guillou H., Miranda G., Pelissier J.-P. (1991). Hydrolysis of b-casein by gastric proteases. Int. J. Peptide Protein Res..

[25] Lopesa A.R., Juliano M.A., Marana S.R., Juliano L., Terra W.R. (2006). Substrate specificity of insect trypsins and the role of their subsites in catalysis. Insect Biochem. Mol. Biol..

[26] Šlechtová T., Gilar M., Kalíková K., Tesařová E. (2015). Insight into trypsin miscleavage: Comparison of kinetic constants of problematic peptide sequences. Anal. Chem..

[27] Vorob’ev M.M., Goncharova I.A. (1998). Computer simulation of proteolysis. Peptic hydrolysis of partially demasked-Lactoglobulin. Nahrung-Food.

[28] Butre C.I., Sforza S., Wierenga P.A., Gruppen H. (2015). Determination of the influence of the pH of hydrolysis on enzyme selectivity of Bacillus licheniformis protease towards whey protein isolate. Int. Dairy J..

[29] Nagy C., Szabo R., Gaspar A. (2022). Microfluidic immobilized enzymatic reactors for proteomic analyses—Recent developments and trends (2017–2021). Micromachines.

[30] Mao Y., Krischke M., Kulozik U. (2019). β-lactoglobulin hydrolysis by immobilized trypsin in ethanol/aqueous solvents. Process Biochem..

[31] Bornhorst G.M., Gouseti O., Wickham M.S.J., Bakalis S. (2016). Engineering digestion: Multiscale processes of food digestion. J. Food Sci..

[32] Acevedo-Fani A., Singh H. (2021). Biopolymer interactions during gastric digestion: Implications for nutrient delivery. Food Hydrocoll..

[33] Farrell H.M., Jimenez-Flores R., Bleck G.T., Brown E.M., Butler J.E., Creamer L.K., Hicks C.L., Hollar C.M., Ng-Kwai-Hang O.F., Swaisgood T.H.E. (2004). Nomenclature of the proteins of cows’ milk, 6th rev. J. Dairy Sci..

[34] Hambling S.G., McAlpine A.S., Sawyer L., Fox P.F. (1992). β-Lactoglobulin. Advanced Dairy Chemistry: Volume 1A: Proteins.

[35] Creamer L.K., Parry D.A., Malcolm G.N. (1983). Secondary structure of bovine beta-lactoglobulin B. Arch. Biochem. Biophys..

[36] Rama G.R., Saraiva Macedo Timmers L.F., Volken de Souza C.F. (2024). In silico strategies to predict anti-aging features of whey peptides. Mol. Biotechnol..

[37] Fox P.F., McSweeney P.L.H. (2003). Advanced Dairy Chemistry—Volume 1: Proteins (Parts A and B).

[38] Grosclaude F., Mahé M.F., Ribadeaudumas B. (1973). Primary structure of alpha casein and of bovine beta casein. Eur. J. Biochem..

[39] Holt C. (1992). Structure and stability of bovine casein micelles. Adv. Protein Chem..

[40] Shapira A., Assaraf Y.G., Livney Y.D. (2010). Beta-casein nanovehicles for oral delivery of chemotherapeutic drugs. Nanomedicine NBM.

[41] McClements D.J., Decker E.A., Park Y., Weiss J. (2009). Structural design principles for delivery of bioactive components in nutraceuticals and functional foods. Crit. Rev. Food Sci. Nutr..

[42] Nongonierma A.B., FitzGerald R.J. (2018). Enhancing bioactive peptide release and identification using targeted enzymatic hydrolysis of milk proteins. Anal. Bioanal. Chem..

[43] Nielsen S.D.H., Liang N., Rathish H., Kim B.J., Lueangsakulthai J., Koh J., Qu Y., Schulz H.J., Dallas D.C. (2023). Bioactive milk peptides: An updated comprehensive overview and database. Crit. Rev. Food Sci. Nutr..

[44] Chobert J.-M., Briand L., Tran V., Haertle T. (1998). How the substitution of K188 of trypsin binding site by aromatic amino acids can influence the processing of b-casein. Biochem. Biophys. Res. Commun..

[45] Leeb E., Stefan T., Letzel T., Hinrichs J., Kulozik U. (2020). Tryptic hydrolysis of b-lactoglobulin: A generic approach to describe the hydrolysis kinetic and release of peptides. Int. Dairy J..

[46] Vreeke G.J.C., Vincken J.-P., Wierenga P.A. (2023). The path of proteolysis by bovine chymotrypsin. Food Res. Int..

[47] Mamone G., Picariello G., Caira S., Addeo F., Ferranti P. (2009). Analysis of food proteins and peptides by mass spectrometry-based techniques. J. Chrom. A.

[48] Vreeke G.J.C., Lubbers W., Vincken J.-P., Wierenga P.A. (2022). A method to identify and quantify the complete peptide composition in protein hydrolysates. Anal. Chim. Acta.

[49] Fernandez A., Riera F. (2013). b-Lactoglobulin tryptic digestion: A model approach for peptide release. Biochem. Eng. J..

[50] Butré C.I., Buhler S., Sforza S., Gruppen H., Wierenga P.A. (2015). Spontaneous, non-enzymatic breakdown of peptides during enzymatic protein hydrolysis. Biochim. Biophys. Acta-Proteins Proteom..

[51] Vorob’ev M.M., Paskonova E.A., Vitt S.V., Belikov V.M. (1986). Kinetic description of proteolysis. Part 2. Substrate regulation of peptide bond demasking and hydrolysis. Liquid chromatography of hydrolyzates. Nahrung-Food.

[52] Vreeke G.J.C., Vincken J.-P., Wierenga P.A. (2023). Quantitative peptide release kinetics to describe the effect of pH on pepsin preference. Proc. Biochem..

[53] Dubois V., Nedjar-Arroume N., Guillochon D. (2005). Influence of pH on the appearance of active peptides in the course of peptic hydrolysis of bovine haemoglobin. Prep. Biochem. Biotechnol..

[54] Sanchez-Reinoso Z., Cournoyer A., Thibodeau J., Said L.B., Fliss I., Bazinet L., Mikhaylin S. (2021). Effect of pH on the antimicrobial activity and peptide population of pepsin hydrolysates derived from bovine and porcine hemoglobins. ACS Food Sci. Technol..

[55] Linderstrøm-Lang K.U. (1952). Lane Medical Lectures.

[56] Vorob’ev M.M., Levicheva I.Y., Belikov V.M. (1996). Kinetics of the initial stages of the hydrolysis of milk proteins by chymotrypsin. Appl. Biochem. Microbiol..

[57] Sopade P.A. (2024). Computational characteristics of kinetic models for *in vitro* protein digestion: A review. J. Food Eng..

[58] Le Feunteun S., Verkempinck S., Floury J., Janssen A., Kondjoyan A., Marze S., Grauwet T. (2021). Mathematical modelling of food hydrolysis during in vitro digestion: From single nutrient to complex foods in static and dynamic conditions. Trends Food Sci. Technol..

[59] Margot A., Flaschel E., Renken A. (1997). Empirical kinetic models for tryptic whey-protein hydrolysis. Process Biochem..

[60] Martinez-Araiza G., Castano-Tostado E., Amaya-Llano S.L., Regalado-Gonzalez C., Martinez-Vera C., Ozimek L. (2012). Modeling of enzymatic hydrolysis of whey proteins. Food Bioprocess Technol..

[61] Valencia P., Pinto M., Almonacid S. (2014). Identification of the key mechanisms involved in the hydrolysis of fish protein by Alcalase. Process Biochem..

[62] Valencia P., Espinoza K., Astudillo-Castro C., Salazar F. (2022). Modeling tool for studying the influence of operating conditions on the enzymatic hydrolysis of milk proteins. Foods.

[63] Beaubier S., Framboisier X., Fournier F., Galet O., Kapel R. (2021). A new approach for modelling and optimizing batch enzymatic proteolysis. Chem. Eng. J..

[64] Marquez M.C., Fernandez V. (1993). Enzymic hydrolysis of vegetable proteins: Mechanism and kinetics. Process Biochem..

[65] Gonzalez-Tello P., Camacho F., Jurado E., Paez M.P., Guadix E.M. (1994). Enzymatic hydrolysis of whey proteins. I. Kinetic model. Biotechnol. Bioeng..

[66] Marquez M.C., Vazquez M.A. (1999). Modeling of enzymatic protein hydrolysis. Process Biochem..

[67] Vorob’ev M.M. (2013). Quantification of two-step proteolysis model with consecutive demasking and hydrolysis of peptide bonds using casein hydrolysis by chymotrypsin. Biochem. Eng. J..

[68] Vorob’ev M.M. (2023). Modeling of the peptide release during proteolysis of β-lactoglobulin by trypsin with consideration of peptide bond demasking. Int. J. Mol. Sci..

[69] Vorob’ev M.M., Butré C.I., Sforza S., Wierenga P.A., Gruppen H. (2016). Demasking kinetics of peptide bond cleavage for whey protein isolate hydrolysed by *Bacillus licheniformis* protease. J. Mol. Catal B.

[70] Rivera-Burgos D., Regnier F.E. (2013). Disparities between immobilized enzyme and solution based digestion of transferrin with trypsin. J. Sep. Sci..

[71] Melikishvili S., Dizon M., Hianik T. (2021). Application of high-resolution ultrasonic spectroscopy for real-time monitoring of trypsin activity in β-casein solution. Food Chem..

[72] Vorob’ev M.M., Vogel V., Güler G., Mäntele W. (2011). Monitoring of demasking of peptide bonds during proteolysis by analysis of the apparent spectral shift of intrinsic protein fluorescence. Food Biophys..

[73] Vorob’ev M.M., Strauss K., Vogel V., Mäntele W. (2015). Demasking of peptide bonds during tryptic hydrolysis of β-casein in the presence of ethanol. Food Biophys..

[74] Adler-Nissen J. (1986). Enzymatic Hydrolysis of Food Proteins.

[75] Rutherfurd S.M. (2010). Methodology for determining degree of hydrolysis of proteins in hydrolysates: A review. J. AOAC Int..

[76] Güler G., Vorob’ev M.M., Vogel V., Mäntele W. (2016). Proteolytically-induced changes of secondary structural protein conformation of bovine serum albumin monitored by Fourier transform infrared (FT-IR) and UV-circular dichroism spectroscopy. Spectrochim. Acta A Mol. Biomol. Spectrosc..

[77] Kafle B., Måge I., Wubshet S.G., Dankel K., Cattaldo M., Böcker U., O’Farrell M., Afseth N.K. (2024). From laboratory to industrial use: Understanding process variation during enzymatic protein hydrolysis with dry film fourier-transform infrared spectroscopy. Food Control.

[78] Buckin V., Altas M.C. (2017). Ultrasonic monitoring of biocatalysis in solutions and complex dispersions. Catalysts.

[79] Dizon M., Buckin V. (2023). Ultrasonic monitoring of enzymatic hydrolysis of proteins. 1. Effects of ionization. Food Hydrocol..

[80] Dizon M., Buckin V. (2025). Ultrasonic monitoring of enzymatic hydrolysis of proteins. 2. relaxation effects. Food Hydrocol..

[81] Dizon M., Tatarko M., Hianik T. (2020). Advances in analysis of milk proteases activity at surfaces and in a volume by acoustic methods. Sensors.

[82] Lotte K., Plessow R., Brockhinke A. (2004). Static and time-resolved fluorescence investigations of tryptophan analogues—A solvent study. Photochem. Photobiol. Sci..

[83] Vivian J.T., Callis P.R. (2001). Mechanisms of tryptophan fluorescence shifts in proteins. Biophys. J..

[84] Quentmeier S., Quentmeier C.C., Walla P.J., Gericke K.-H. (2009). Two-color two-photon excitation of intrinsic protein fluorescence: Label-free observation of proteolytic digestion of bovine serum albumin. ChemPhysChem.

[85] Karuso P., Crawford A.S., Veal D.A., Scott G.B.I., Choi H.-Y. (2008). Real-time fluorescence monitoring of tryptic digestion in proteomics. J. Proteome Res..

[86] Zeng J., Zou J., Zhao J., Lin K., Zhang L., Yi H., Gong P. (2023). Chymosin pretreatment accelerated papain catalysed hydrolysis for decreasing casein antigenicity by exposing the cleavage site at tyrosine residues. Food Chem..

[87] Vorob’ev M.M. (2022). Modeling of proteolysis of β-lactoglobulin and β-casein by trypsin with consideration of secondary masking of intermediate polypeptides. Int. J. Mol. Sci..

[88] Vorob’ev M.M. (2019). Proteolysis of β-lactoglobulin by trypsin: Simulation by two-step model and experimental verification by intrinsic tryptophan fluorescence. Symmetry.

[89] Vorob’ev M.M., Açıkgöz B.D., Güler G., Golovanov A.V., Sinitsyna O.V. (2023). Proteolysis of micellar β-casein by trypsin: Secondary structure characterization and kinetic modeling at different enzyme concentrations. Int. J. Mol. Sci..

[90] Vorob’ev M.M. (2020). Tryptophan fluorescence and time-lag hydrolysis of peptide bonds during degradation of β-lactoglobulin by trypsin. Catalysts.

[91] Creusot N., Gruppen H. (2007). Hydrolysis of whey protein isolate with Bacillus licheniformis protease: Fractionation and identification of aggregating peptides. J. Agric. Food Chem..

[92] Creusot N., Gruppen H. (2008). Hydrolysis of whey protein isolate with Bacillus licheniformis protease: Aggregating capacities of peptide fractions. J. Agric. Food Chem..

[93] Cheison S.C., Leeb E., Toro-Sierra J., Kulozik U. (2011). Influence of hydrolysis temperature and pH on the selective hydrolysis of whey proteins by trypsin and potential recovery of native alpha-lactalbumin. Int. Dairy J..

[94] Deng Y., Butré C.I., Wierenga P.A. (2018). Influence of substrate concentration on the extent of protein enzymatic hydrolysis. Int. Dairy J..

[95] Hinnenkamp C., Ismail B.P. (2021). A proteomics approach to characterizing limited hydrolysis of whey protein concentrate. Food Chem..

[96] Li S., Carne A., Bekhit A.E.-D.A. (2024). Investigation of antioxidant activity of protein hydrolysates from New Zealand commercial low-grade fish roes. Mar. Drugs.

[97] Vorob’ev M.M., Sinitsyna O.V. (2020). Degradation and assembly of β-casein micelles during proteolysis by trypsin. Int. Dairy J..

[98] Vorob’ev M.M., Vogel V., Mäntele W. (2013). Demasking rate constants for tryptic hydrolysis of β-casein. Int. Dairy J..

[99] Golovanov A., Güler G., Vorob’ev M.M. (2023). Modification of protein micelles by limited hydrolysis of peptide bonds: A model of the sequential degradation of β-casein micelles. INEOS OPEN.

[100] Rogers M.A. (2024). The role of food structure in the biophysics of digestion: The remarkable coevolution of the casein micelle. Food Biophys..

[101] Bing H., Hong X., Tao X., Liu D., Zhang J., Yang T., Liu T.C., Liu X., Zhou P. (2024). Structure and digestibility of bovine casein complexes formed by enriching k- and b-caseins in micellar casein concentrate together with minerals adjustment. Int. Dairy J..

